# A uniform and versatile surface‐guided radiotherapy procedure and workflow for high‐quality breast deep‐inspiration breath‐hold treatment in a multi‐center institution

**DOI:** 10.1002/acm2.13511

**Published:** 2022-01-20

**Authors:** Guang Li, Wei Lu, Kyle O'Grady, Iris Yan, Ellen Yorke, Laura I Cervino Arriba, Simon Powell, Linda Hong

**Affiliations:** ^1^ Department of Medical Physics Memorial Sloan Kettering Cancer Center New York New York USA; ^2^ Department of Radiation Oncology Memorial Sloan Kettering Cancer Center New York New York USA

**Keywords:** deep‐inspiration breast hold (DIBH), left‐sided breast radiotherapy, optical surface imaging (OSI), surface‐guided radiotherapy (SGRT)

## Abstract

**Purpose:**

We share our experiences on uniformly implementing an effective and efficient SGRT procedure with a new clinical workflow for treating breast patients in deep‐inspiration breath‐hold (DIBH) among 9 clinical centers using 26 optical surface imaging (OSI) systems.

**Methods:**

Our procedures have five major components: (1) acquiring both free‐breathing (FB) and DIBH computed tomography (CT) at simulation to quantify the rise of the anterior surface, (2) defining uniformly a large region of interest (ROI) to accommodate large variations in patient anatomy and treatment techniques, (3) performing two‐step setup in FB by first aligning the arm and chin to minimize breast deformation and reproduce local lymphnode positions and then aligning the ROI, (4) aligning the vertical shift precisely from FB to DIBH, and (5) capturing a new on‐site reference image at DIBH to separate residual setup errors from the DIBH motion monitoring uncertainties. Moreover, a new clinical workflow was developed for patient data preparation using 4 OSI offline workstations without interruption of SGRT treatment at 22 OSI online workstations. This procedure/workflow is suitable for all photon planning techniques, including 2‐field, 3‐field, 4‐field, partial breast irradiation (PBI), and volumetric‐modulated arc therapy (VMAT) with or without bolus.

**Results:**

Since 2019, we have developed and applied the uniform breast SGRT DIBH procedure with optimized clinical workflow and ensured treatment accuracy among the nine clinics within our institution. About 150 breast DIBH patients are treated daily and two major upgrades are achieved smoothly throughout our institution, owing to the uniform and versatile procedure, adequate staff training, and efficient workflow with effective clinical supports and backup strategies.

**Conclusion:**

The uniform and versatile breast SGRT DIBH procedure and workflow have been developed to ensure smooth and optimal clinical operations, simplify clinical staff training and clinical troubleshooting, and allow high‐quality SGRT delivery in a busy multi‐center institution.

## 1 INTRODUCTION

Surface‐guided radiation therapy (SGRT) for left‐sided breast deep‐inspiration breath‐hold (DIBH) treatment using optical surface imaging (OSI) has been applied in the clinic for years^1^, ^2^, ^3^, ^4^, ^5^, ^6^ to spare the heart from radiation. Historically, the conventional spirometer,^7^, ^8^ active breathing control (ABC),^9^, ^10^ and real‐time position management (RPM)^11^, ^12^ system have served as different surrogates to monitor the level of patient's breath‐hold. Recently, SGRT has been introduced to replace these surrogates with the advantage of three‐/four‐dimensional (3D/4D) surface guidance for both patient setup and DIBH gating.

A widely used OSI system is the AlignRT^®^ system (VisionRT, London, UK), which contains three ceiling‐mounted camera pods (two lateral and one frontal) and each pod hosts two cameras providing a stereoscopic view of patients’ body surface. Using structured light with a speckle pattern, the point array on the surface can be identified by both cameras for the reconstruction of a 3D surface image^13^ and combining the three surfaces from the three‐camera pods, a large 3D body surface image is provided covering a large (∼1 m) field of view (FOV) for patient setup.^14^ A region of interest (ROI) on the 3D surface of a patient is defined on the external body contour of the simulation computed tomography (CT) image and matched with the OSI image using vendor's proprietary surface registration based on the least root‐mean‐square (RMS) error algorithm to achieve SGRT setup.

At a lowered spatial resolution, real‐time reconstruction of the ROI surface can be realized with a reduced number of points in the surface point array, and a real‐time delta (RTD) mode can be applied to provide real‐time ROI surface matching (4–12 Hz) for monitoring patient motion during setup and treatment. Additionally, the AlignRT system can communicate with a linear accelerator (Linac) through the motion management interface (MMI) to apply SGRT shifts to the treatment couch and to gate the radiation by holding a beam if a patient's motion exceeds a set tolerance and resuming it when the motion falls back into the tolerance.

In our institution with 9 clinical centers, 26 AlignRT systems are currently employed, including 22 online workstations with the MMI enabled for treatment and 4 offline workstations for patient data preparation. They are primarily used to guide breast DIBH setup and treatments and brain stereotactic radiosurgery (SRS) and radiotherapy (SRT). As one of the largest users of the AlignRT technology, we are among the first to encounter the challenge of managing a large number of clinical AlignRT systems for SGRT treatments. The major advantages of SGRT include a large FOV for initial patient setup and intra‐fractional motion monitoring in real‐time throughout treatment. However, the surface surrogate also adds complexity during planning and treatment, including ROI definition, tissue deformation, and extra workload. In our institution, radiotherapy planning is centralized, meaning that a dosimetrist from any clinic does plans for all clinics within the institution, regardless of physical locations. Therefore, a uniform, versatile, and easy‐to‐follow procedure needs to be established so that complex treatment operations can be executed with high and consistent quality in patient care throughout our clinical network.

In this study, we report a uniform and versatile breast DIBH SGRT procedure and workflow that can minimize breast deformation, ensure the reproducible position of the local lymph nodes, and monitor the vertical shift precisely from free‐breathing (FB) to DIBH regardless of whether bolus is used for treatment. This procedure has combined the knowledge from breast DIBH SGRT publications,^1^, ^2^, ^3^, ^4^, ^5^, ^6^ as well as our clinical experiences on breast SGRT setup in FB since 2012,^15^, ^16^, ^17^, ^18^ SGRT for brain SRS/SRT since 2010,^19^, ^20^ and RPM‐guided DIBH technique since 2002.^21^ We discuss several major clinical and operational considerations and solutions in the breast SGRT DIBH procedure and workflow, including patient data preparation, clinical staff training, and clinical benefits.

## 2 MATERIALS AND METHODS

### 2.1 Twenty‐six AlignRT systems in multi‐center clinics

Twenty‐six AlignRT systems (versions 5.1 and 6.2) in nine clinics within our institution are commissioned for clinical applications, including four offline workstations, dedicated for patient data preparation during treatment hours when the online workstations located at the treatment console are occupied. They account for about 2/3 of the treatment Linacs (19 TrueBeam and 3 Trilogy, Varian Medical Systems, Palo Alto, CA). The MMI communication is enabled between AlignRT and TB Linac systems for automatic couch shifting and beam gating. All 22 online treatment AlignRT systems are connected to the Eclipse planning system (Varian Medical Systems, Palo Alto, CA) via either the offline workstation (v5.1) or AlignRT server (v6.2) using the digital communication in medicine (DICOM) protocol. The four AlignRT offline workstations are shared by nine clinics to allow dosimetrists to prepare new patient data in either v5.1 or v6.2 (see discussion in the next section), while the online workstations are dedicated for the patient treatment during the day.

Three major SGRT workflows and procedures have been developed and used in our multi‐center clinics, including breast FB setup, breast DIBH treatment, and brain SRS/SRT treatment. These procedures are simplified and unified with similar operations. For breast SGRT DIBH setup and treatment, both FB and DIBH CT images are acquired at simulation in order to quantify the position difference of the anterior surface from the FB to DIBH setups. As a patient plan is assigned to a dosimetrist regardless of where the patient is to be treated within our institution, it is essential to develop a uniform AlignRT preparation procedure and workflow for the planning process using various planning techniques.

Although about two‐thirds of all linacs are equipped with AlignRT systems in our institution, allowing a backup strategy by moving patients among AlignRT‐equipped linacs, we still allow RPM gating as the additional backup method (the primary method on non‐AlignRT machines) by checking the box of “use gated” for all breast SGRT DIBH plans, so the same plan can be delivered using either AlignRT or RPM for DIBH gating.

### 2.2 The uniform workflow for patient preparation during regular MMI operation hours

As the MMI is enabled for all AlignRT‐Linac systems, all AlignRT online workstations are in use regardless of treating AlignRT patients or non‐AlignRT patients (in auto‐authorization mode). Therefore, these workstations are not available to prepare patient data (PData) for new patients during working hours. To overcome this limitation, we have developed a new clinical workflow by using four AlignRT offline workstations dedicated to PData preparation at any time. These workstations are shared by our dosimetrists via local or remote access, roughly one offline workstation per 2 clinics, to feed the 22 AlignRT online treatment systems in 9 clinical centers. In v5.1, we developed an in‐house program, Dispatcher, to distribute the prepared PData to all AlignRT treatment systems within a clinic and perform basic computerized QA.^22^ The four offline workstations were set as the DICOM export destinations from Eclipse, used to prepare, dispatch, and QA PData. In v6.2, the AlignRT application server is available as the DICOM destination for the Eclipse export filter and links the centralized Structured Query Language (SQL) database and the front‐end AlignRT offline and online workstations. So, the prepared PData on an offline workstation can be accessed by all online workstations for treatment through the servers.

The ROI definition, used for patient setup and motion monitoring, is a critical component of patient preparation. It is key to create a uniform ROI definition to simplify SGRT preparation for all patients with different sizes and shapes in multi‐center clinics so that the same or similar treatment quality can be ensured. For a robust breast SGRT DIBH treatment procedure, a large ROI is needed, which is defined with four boundaries: from the body midline in the sagittal view on the ipsilateral side to the nipple of the contralateral breast in the medial–lateral direction and from the supraclavicular match‐line to 2 cm below the breast tissue in the superior–inferior direction. This large ROI definition is for all whole‐breast treatments, regardless of planning/treatment methods to ensure (1) to include the stable area in the mediastinum surface for accuracy, (2) to contain an asymmetrical ROI to eliminate the potential ambiguity in surface matching, and (3) to provide sufficient ROI surface for detection so that the surface monitoring will be less vulnerable to gantry blocking of camera views during volumetric‐modulated arc therapy (VMAT) treatment.^23^ For ∼5% of patients, the ROI needs to be further enlarged to include the entire contralateral breast to overcome the gantry‐blocking problem, and therapists are given the right for online ROI modification. For partial‐breast irradiation (PBI) DIBH cases, the ROI is smaller for better tumor‐bed localization and alignment, including only the ipsilateral breast with a 2 cm margin.

### 2.3 The uniform patient treatment procedure

In SGRT FB setup, a two‐step setup procedure is applied: (1) to align the arm and chin first by adjusting the patient, guided by the in‐room AlignRT surface‐matching display, and (2) to align the FB ROI by applying couch shifts. In both steps, the DICOM body contour references are used for the in‐room patient SGRT FB setup, which usually takes less than 2 min. The large FOV of OSI made arm and chin adjustment possible with static (v5.1) or real‐time (v6.2) visual guidance, in contrast to the limited kV imaging view. Reproducing the arm/chin positions from simulation minimizes the deformation of the breast and reproduces the positions of local lymph nodes,^24^ critical to treatment quality. After the FB setup, the patient is instructed to take a DIBH to match the DIBH DICOM ROI reference. The vertical shift of the surface is monitored closely during DIBH. Once the DIBH setup is satisfactory, a new on‐site reference surface image is acquired for DIBH motion monitoring regardless of bolus application so that the residual SGRT setup errors are separated from the SGRT motion monitoring uncertainties. When a bolus is prescribed, it is applied quickly within a DIBH and a new reference is then captured. This is also consistent with the brain SRS/SRT treatment procedure. Therefore, regardless of the treatment sites, an on‐site reference is always acquired after patient setup and used for motion monitoring, simplifying the clinical operations.

From FB to DIBH, the most critical parameter is the anterior–posterior (AP) position of the ROI surface, because it serves as a surrogate for chest wall separation from the heart. After a patient is set up in FB, the couch's vertical height (in the AP direction) is kept unchanged while lateral and longitudinal couch adjustments are allowed during DIBH. This prevents the situations where the patient's breath‐hold is at a shallower (or deeper) level, but the couch height is raised (or lowered) to generate an untruthful match. As inherited from the previous RPM‐guided DIBH procedure, the DIBH tolerance is kept at ±3 mm, while ±5 mm is allowed for patients who have difficulty reproducing the simulation DIBH position. The loosen threshold can still be beneficial to reduce heart dose, achieving a partial heart sparing effect.^25^, ^26^

**FIGURE 1:**
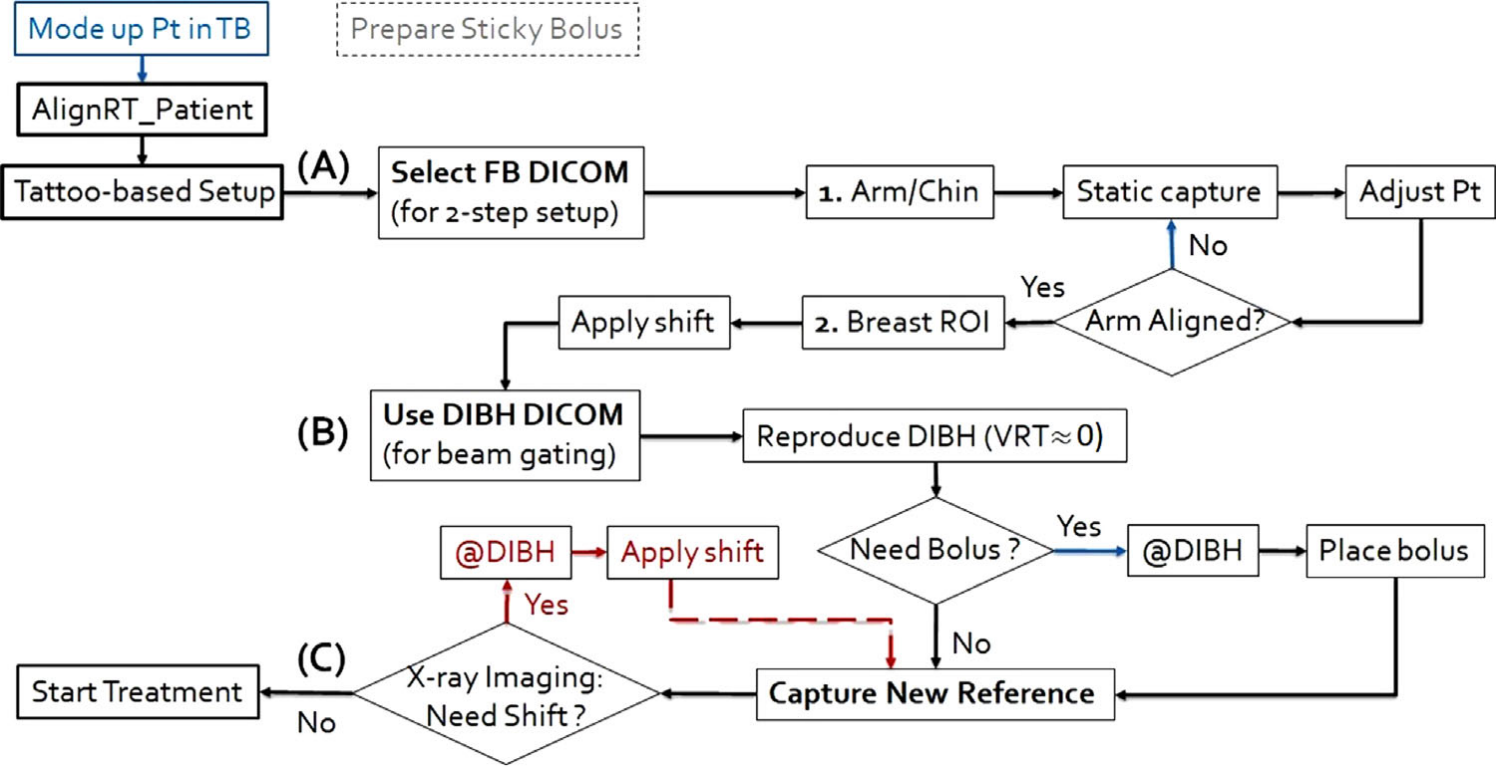
A uniform clinical workflow for surface‐guided radiotherapy (SGRT) treatment for breast cancer patients using the deep‐inspiration breath‐hold (DIBH) method. (a) First setup the patient at free‐breathing (FB) using the two‐step setup strategy to align the arm/chin first and then breast region of interest (ROI), (b) setup patient at DIBH, including the option of bolus placing and X‐ray imaging, and (c) deliver the treatment at DIBH with the motion management interface (MMI) for beam control. Note that a new on‐site reference is always captured regardless of imaging or bolus conditions before DIBH treatment

To simplify the SGRT DIBH treatment, a uniform procedure is developed and used in all the clinics for all treatment techniques, such as 2F/3F/4F, PBI butterfly 4F, and locally‐advanced breast VMAT, with or without bolus application, and with or without daily 2DkV imaging, as shown in Figure 1. Note that the sticky bolus (Radiation Products Design, Inc.) is used with an outer white‐cloth surface that can be imaged by the OSI and an inner sticky surface to the skin. For 3F/4F SGRT DIBH treatment, the sticky bolus is placed at setup to align the superior edge on the super‐clavicle (SCV) match‐line, so both tangents and SCV fields are delivered without entering the room. Note that two DIBH reference images are acquired for tangents and the SCV treatments. For VMAT DIBH treatments,^23^ a bolus (3–10 mm thickness) and daily 2DkV are always prescribed, providing a direct comparison between SGRT and image‐guided radiotherapy (IGRT) setups at DIBH.

## 3 RESULTS

### 3.1 Uniform clinical workflow for AlignRT patient preparation using offline workstations

Introducing 4 offline workstations to support clinical patient preparation and treatment for 22 AlignRT systems has been successful for more than 2 years using either AlignRT v5.1 and v6.2. The Dispatcher program was developed and used to support our clinical operation for 2 years before the completion of the v6.2 upgrade in 2021. Not only can patient data be prepared during treatment hours, but they are checked automatically for patient information, plan parameters, and past and existing treatments as well. It also allows manual checking as it extracts the DICOM information. In v6.2, the same workflow still works with the centralized SQL‐DB to host the patient data, which are accessible via the AlignRT application server by all online workstations within our institution for treatment.

### 3.2 Uniform ROI definitions for whole‐breast and partial‐breast irradiations

**FIGURE 2:**
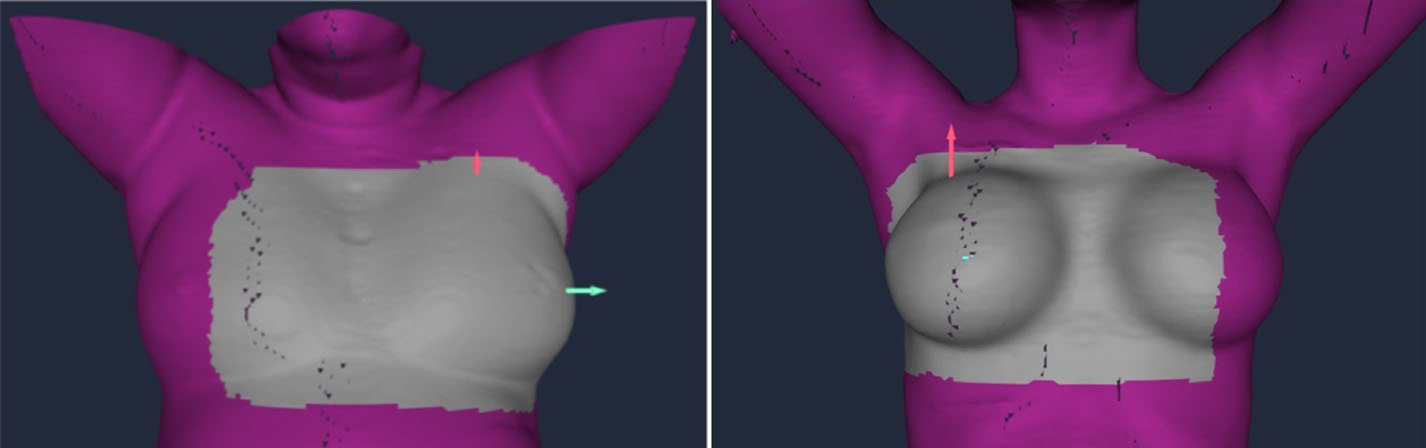
Breast region of interest (ROI) definitions for whole‐breast treatments: (a) the ROI in left‐sided native breast treatment and (b) the ROI for right‐sided implanted breast treatment. The breast deep‐inspiration breath‐hold (DIBH) surface‐guided radiotherapy (SGRT) procedure is mostly applied to treat left‐sided breast patients but is also used to treat right‐sided breast patients in our institution

The ROI definition for whole breast treatments is the same regardless of planning methods. Figure 2 shows two typical ROI examples, covering both the ipsilateral breast and half of the contralateral breast. However, for PBI treatments, the ROI is defined differently, covering only the ipsilateral breast with a 2 cm margin.

### 3.3 Minimizing breast deformation by reproducing the ipsilateral arm position

**FIGURE 3:**
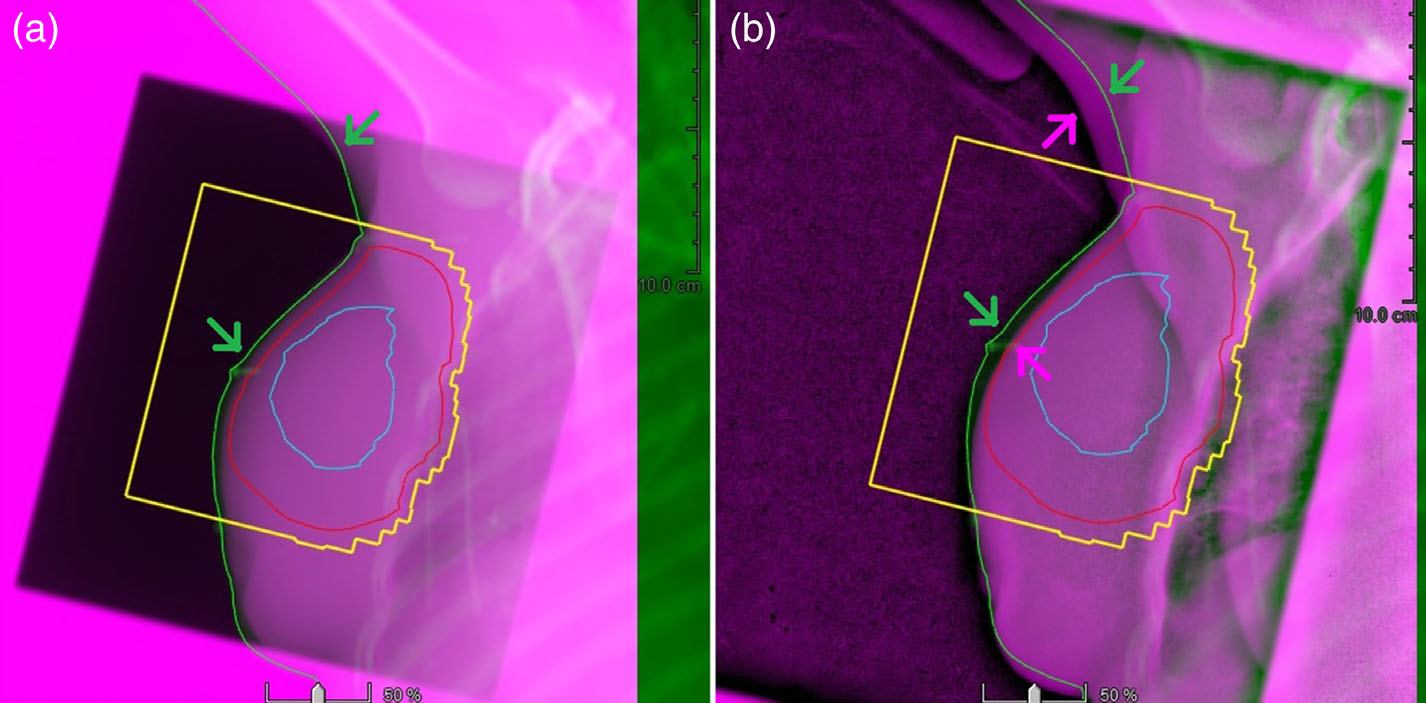
An example of breast deformation caused by arm position variation for a partial breast irradiation (PBI) patient. The tumor‐bed volume (blue), planning tumor volume (red), beam aperture (yellow), and simulation body contour (green) are shown. (a) A good arm alignment allows a good breast alignment between the setup portal image (pink) and the body contour (green) in the digitally reconstructed radiograph (DRR) of simulation computed tomography (CT) and (b) somewhat deformed breast near the tumor bed (green and pink arrows in b) when the arm (green and pink arrows in b) is misaligned by ∼1 cm in the beam eye's view

Figure 3 illustrates a PBI case, in which a good arm alignment minimizes breast deformation, whereas misalignment in the arm results in noticeable breast deformation in the portal images. The SGRT adjustment of the patient's arm/chin is achieved by moving the patient with real‐time OSI guidance. As different arm positions result in different stretches of the pectoralis major muscle that causes breast deformation with different extents, reproducing the arm position at simulation should minimize the breast deformation at treatment.

### 3.4 Minimizing position variations of local lymph nodes by reproducing the ipsilateral arm

**FIGURE 4:**
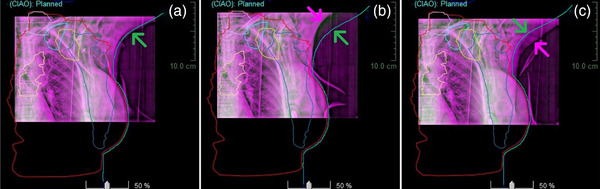
Illustration of the arm alignment of a locally advanced breast patient with implanted breast in volumetric‐modulated arc therapy deep‐inspiration breath‐hold (VMAT DIBH) setup, in which the simulation body contour (green) is overlaid in the image fusion between the setup anterior–posterior (AP, pink) and planning digitally reconstructed radiograph (DRR, green) images. (a) A good arm alignment, (b) medially arm misalignment, and (c) laterally arm misalignment, as indicated by the arrows (pink and green). The planning tumor volume (PTV) (red) and local lymph nodes from planning computed tomography (CT) are also overlaid to the planning DRR, including axillary nodes level I (blue), II (yellow), and III (light blue), super clavicle node (gray), and internal mammary node (badge). The nodes within the PTV would be deformed and changed if the arm is away from the simulation position (green)

Figure 4 demonstrates position variations of the five breast lymph nodes in AP kV radiographic images due to different arm positioning at treatment setup. When the arm is off alignment by 1–3 cm laterally in the same superior–inferior level of the lymph nodes around the clavicle region, the axillary nodes (level I, II, and III) can be off accordingly, owning to stretching of the pectoralis major muscle from the arm's lateral edge to the medial center of the body. For locally advanced breast patients, as shown in this case, the local nodes are part of the planning tumor volume (PTV), so it could generate a large setup uncertainty in the nodal targets, although the breast seems aligned well for this patient with implanted breasts. Therefore, it is necessary to perform the two‐step SGRT breast setup to avoid large arm misalignment that could lead to suboptimal treatment, especially for VMAT treatments.^17^

### 3.5 Examples of DIBH vertical position difference between AlignRT and 2DkV

Table 1 tabulates a statistical comparison between AlignRT and 2DkV of 25 patients in their DIBH setup for VMAT treatments (200cGy x 25) of locally advanced breast cancer. For these patients, daily 2DkV and bolus are prescribed for VMAT treatments with DIBH. The average differences and standard deviations of vertical shifts are provided with pitch rotations since they have minor correlation.^27^ Despite anatomic variations of the breast patients, from small to pendulous and from mastectomy to implants, as shown in Table 1, the DIBH setup differences are relatively small (1.3±1.7 mm) between SGRT (aligning on the anterior body surface) and IGRT (aligning on the anterior bony landmark).

**TABLE 1:** Comparison of vertical shift (with pitch rotation) difference between AlignRT and 2DkV for locally advanced breast DIBH SGRT patients treated with VMAT with a bolus in 25 patients, who had various breast conditions and were treated across the multi‐center clinics. The AlignRT setup was done first, followed by 2DkV, therefore, the 2DkV represent the difference from the AlignRT setup. The anterior surface distances at the inferior sternum from FB to DIBH are also listed, quantifying the surface shift in the anterior–posterior (AP) direction

	**Patient conditions**			**Vertical (cm)**		**Pitch (˚)**		**AP distance**
**Patient**	**Age**	**Treated breast**	**PTV** **^#^** **( cm 3 )**	**Mean**	**St Dev**	**Mean**	**St Dev**	**DIBH‐FB (cm)**
1	50	Mastectomy	1321	−0.08	0.26	−0.20	0.66	0.97
2	43	Bilateral implant	1631	0.10	0.42	0.20	1.28	0.46
3	56	Intact, medium	1086	0.04	0.19	0.52	0.80	1.70
4*	59	Bilateral implant	1657	−0.44	0.44	−0.82	1.02	1.25
5	43	Lt implant only	1132	−0.05	0.26	0.29	0.83	0.85
6	31	Bilateral implant	1733	−0.35	0.75	0.44	1.11	1.18
7	36	Bilateral implant	2187	−0.15	0.41	−0.19	0.72	1.07
8	43	Intact, pendulous	2596	−0.14	0.38	−0.25	0.72	0.76
9	67	Intact, large	1974	−0.22	0.31	−0.01	0.24	1.78
10	34	Bilateral implant	1336	−0.35	0.47	−0.27	0.57	0.46
11	43	Bilateral implant	1317	0.03	0.27	−0.17	0.51	1.55
12	49	Mastectomy	1140	0.03	0.27	−0.17	0.51	0.81
13	53	Bilateral implant	2638	−0.29	0.27	0.04	0.67	0.74
14	74	Intact, small	820	−0.31	0.27	0.10	0.74	0.62
15	68	Mastectomy	1016	−0.16	0.46	−0.95	1.14	1.96
16	46	Intact, large	2419	−0.08	0.36	0.05	0.18	1.46
17	38	Bilateral implant	1746	−0.13	0.15	−0.01	0.54	2.45
18	55	Native, large	2500	−0.25	0.24	−0.55	0.81	1.20
19	39	Mastectomy	2099	0.07	0.34	0.39	0.78	0.90
20	34	Lt implant only	1825	−0.29	0.37	−0.72	1.14	1.72
21	65	Native, medium	1639	0.01	0.38	−0.69	0.39	1.55
22	38	Native, large	2448	−0.28	0.16	−0.05	1.00	1.51
23	55	Mastectomy	1012	0.08	0.66	0.07	1.05	1.08
24	36	Bilat implant	1886	0.22	0.40	0.03	0.03	0.55
25	40	Mastectomy	1583	−0.13	0.37	0.54	0.74	0.94
Average	48		1710	−0.13	0.35	−0.10	0.72	1.18
St Dev	12		544	0.17	0.14	0.41	0.32	0.51

^#^ *Notes*: The PTV includes both breast tissue and local lymph nodes.

* The large mean vertical shift is due to the pitch rotation that has a minor correlation with the vertical translation.

Abbreviations: DIBH, deep‐inspiration breath‐hold; FB, free‐breathing; SGRT, surface‐guided radiotherapy; VMAT, volumetric‐modulated arc therapy; PTV, planning tumor volume.

## 4 DISCUSSION

During the development of the breast SGRT DIBH procedure, a team of expert physicists in AlignRT and breast treatment were tasked to combine their clinical knowledge into the clinical procedure, including existing publications from other institutions^1^, ^2^, ^3^, ^4^, ^5^, ^6^ and clinical experience at our institution.^15^, ^16^, ^17^, ^18^, ^19^, ^20^, ^28^ Therefore, the new procedure and workflow are not only unique, uniform, and straightforward for clinical implementation, but also versatile, flexible, long‐lasting to meet our clinical conditions and requirements.

### 4.1 The uniqueness of the uniform breast SGRT DIBH procedure and workflow

First, for all DIBH patients, both FB and DIBH simulation CT images are acquired to allow ample time (within 1–2 min) for patient setup in two steps during FB and quantifying the vertical depth of inspiration from FB to DIBH (Table 1). Therefore, using these simulation CT DICOM surfaces, the DIBH position can be accurately reproduced during treatment under AlignRT guidance. All existing photon planning techniques are suitable for breast SGRT DIBH treatments. The DIBH procedure is also applied to right‐sided breast patients for lung‐sparing occasionally (see Figure 2B).

Second, a uniform ROI definition simplifies clinical operations. For whole‐breast patients, the ROI is large, covering the ipsilateral breast and half of the contralateral breast so that it avoids a symmetric ROI causing ambiguous surface registration, includes the sternum area with minimal deformation, and provides sufficient visible area (> 50% ROI) during VMAT delivery. The ROI is copied from DIBH to FB plan in AlignRT to save preparation time and to keep the consistency in patient vertical alignment.

Third, patient setup in FB allows ample time to achieve the two‐step SGRT setup,^15^ which is to align the arm and chin first, in order to reproduce the positions of the breast and local lymph nodes with minimal deformation and displacement, as illustrated in Figures 2 and 3. This is particularly important for locally advanced patients whose local nodes are treatment targets. The large FOV (∼100 × 100 cm^2^) makes the OSI technique the only imaging modality that can guide therapists to adjust the patient's arm and chin in real‐time. After the arm/chin alignment, the final alignment correction is through manual or automatic couch shifts (via the MMI).

Fourth, from FB to DIBH, the vertical shift is critically monitored as it is directly correlated to the chest wall position. As the ROI is copied and pasted between FB and DIBH, the vertical shift at the ROI provides an accurate assessment of chest wall elevation. The goal of the DIBH setup is to achieve a near‐zero residual shift in the vertical direction using the DICOM DIBH reference (Figure 1), so that the new treatment reference will carry a minimal residual setup error.

Lastly, a new on‐site reference surface is uniformly captured for motion monitoring for all of our clinical SGRT procedures, including breast DIBH treatments, regardless of the use of bolus or not, making clinical implementation and execution much easier. As a new on‐site reference image resets the residual setup uncertainties to zero, it eliminates their interference to the DIBH motion thresholds. Otherwise, the residual shifts (1–2 mm) carried from setup will be accounted as motion, narrowing DIBH tolerance (±3 mm), triggering frequent beam hold, and interfering treatment delivery.

### 4.2 The versatility of the uniform breast SGRT DIBH procedure and workflow

The introduction of four AlignRT offline workstations in our clinics allows AlignRT PData preparation for new patients during treatment hours (with MMI enabled) in both v5.1 and v6.2. The four AlignRT offline workstations allow four simultaneous usages, back up each other in case some are down, and remote login for dosimetrists to complete the AlignRT preparation at their planning workstation. This is especially valuable during the pandemic period as it supports work from home. The redundant offline workstations can also be used to apply different versions to support multi‐center clinics during a staggered system upgrade, which may last weeks or even months, depending on the resources of both the vendor and the multi‐center clinics. During the AlignRT v6.2 upgrade, these four offline workstations were indeed used in different versions to accommodate the speed of the system upgrade for 22 AlignRT online workstations.

All photon planning techniques can be applied in breast SGRT DIBH treatments, including the conventional opposite tangent fields (2F), 3F (2F + SCV) and 4F (3F + posterior axillary boost field (PAB)), accelerated PBI for early‐stage breast cancer, and VMAT to treat locally‐advanced breast cancer. The procedure can also accommodate the use of 2DkV imaging for setup and bolus for treatment, as shown in Figure 1C and Table 1. In our 3F/4F procedure, the bolus is placed at the DIBH setup with the superior edge matched to the SCV match‐line so no need to enter the room for bolus placement during treatment.

The uniform ROI definition for whole‐breast treatment is good for almost all patients; only a few VMAT patients may experience the temporary gantry‐blocking problem during VMAT delivery, resulting in undeterminable patient motion as < 50% ROI is visible. In this case, our clinical solution is to allow therapists to further enlarge the ROI to include the bilateral breasts so that sufficient ROI can be seen for motion detection and the treatment can be resumed. Note that 2F/3F/4F patients do not have this problem, because the ipsilateral camera is never blocked. For patients who experience difficulties to reproduce the DIBH position, we allow increasing the DIBH tolerance threshold from ±3 mm to ±5 mm, which can still preserve most of the heart sparing benefit.^26^ During the DIBH treatment, if the patient goes out of the DIBH threshold, the radiation beam will be automatically held, until the patient position is falling back to within the threshold. If a patient moves her body during treatment beyond 3 mm or 2° in other directions of the 6DOF, the therapist will stop the treatment and verify the patient setup in FB, and redo the patient setup if the movement is out of the clinical tolerance.

### 4.3 Clinical benefits of establishing and following a uniform procedure

The uniform SGRT procedure for breast DIBH treatment in our multi‐center institution has greatly improved the clinical operations, including clinical workflow, staff training, operation precision, and treatment delivery accuracy. The uniformity of the procedure simplifies the clinical operation, while the versatility allows handling various clinical situations.

First, the new clinical procedure and workflow using four offline workstations are essential to use the MMI for automatic couch shift and beam gating, prepare and distribute PData of new patients using in‐house developed Dispatcher program (v5.1), and allows a smooth clinical transition for future upgrades (v6.2 and beyond). Second, The uniformity of the procedure and workflow facilitates the clinical implementation, including the initial and continuous staff training, clinical troubleshooting, as well as experience sharing across centers, as staff training is a challenge in our 9‐center institution to ensure ∼120 dosimetrists and physicists and ∼100 therapists follow the complex procedures precisely. Lastly, and most importantly, the uniformity of the procedure and workflow helps to deliver high‐quality breast DIBH SGRT treatments, as all dosimetrists and therapists can follow the written procedure at different physical locations and deliver nearly uniform performance across all centers within the institution.

It is worthwhile to mention that the physics support to clinical operations is also simplified with sharable experience and becomes more effective and efficient. For instance, we have formed an SGRT team consisting of local AlignRT‐point physicists at each of the nine centers to provide center‐wide clinical support, share clinical experiences, and provide therapist training to handle routine issues. we have also developed a common event log system shared by the SGRT team so that the questions and answers are made available to therapists as troubleshooting tips. As shown in Table 1, AlignRT setup accuracy is acceptable for these randomly‐picked patients with various sizes and shapes. In summary, the uniformity of the procedures facilitates precise clinical operations in both initial implementation and long‐term execution across a multi‐center institution.

Despite the clinical benefits, several limitations and precautions need to be mentioned. The uniform SGRT procedure does not allow electron beams due to the blocking of the ROI view by the electron cone. It also does not apply to prone breast treatment, which is an alternative treatment option to spare the heart for patients without nodal involvement. For patients going through breast mastectomy and implantation during radiotherapy, the implant may be deflated, and therefore patient surface will be different from the DICOM at simulation. In this situation, the RPM‐guided DIBH procedure can be used as a backup. The RPM‐gating backup can also be applied if the AlignRT system is down and if the AlignRT patient data need troubleshooting or being re‐prepared, so the patient can be treated without delay. Alternatively, the patient could be moved to another AlignRT‐linac machine for treatment if AlignRT is down, as seven out of our nine centers are equipped with more than two AlignRT systems. Finally, for patients with difficulties reproducing the simulated DIBH positions, increasing the SGRT gating threshold should be allowed to gain as much heart sparing as possible.

## 5 CONCLUSION

A uniform and versatile SGRT DIBH procedure and workflow have been developed and implemented using 22 AlignRT‐Linac systems and four AlignRT offline workstations in our nine‐center clinics successfully. This SGRT procedure is suitable for all supine photon breast DIBH planning and treatment techniques. It has greatly simplified and facilitated the clinical operation and ensured precise execution of the complex procedure with high‐quality treatment delivery.

## CONFLICT OF INTEREST

The authors declare that there is no conflict of interest that could be perceived as prejudicing the impartiality of the research reported.
